# Orthodontia

**Published:** 1902-10-15

**Authors:** E. H. Angle


### ORTHODONTIA.

Extracts from a paper read by E. H. Angle, M.D., D.D.S., before Tuft's College Alumni Association, and published in the September *Cosmos*.

According to our literature it is now three hundred and fifty-six years since we began the practice of orthodontia.

The establishment of perfect occlusion means the establishment of harmony throughout.

In proportion as malocclusion exists so is the beauty of the face marred, the usefulness of the teeth impaired, and the articulate speech effected.

The practice of orthodontia is woefully neglected, not because there is a lack of opportunity, or lack of appreciation of well directed or intelligent treatment by those needing this kind of service. Among the many reasons may first be noted the fact that educational institutions do not adequately teach it, therefore students do not become interested in it to the extent that they should to become enthusiasts or even intelligent in regard to its method. Other studies are permitted to crowd it aside. But more than all else comparatively few students have the ability in aptitude or liking for the work to ever become sufficiently proficient to practice it successfully. I believe it to be quite as absurd to attempt to teach all dental students orthodontia with the expectation of there becoming proficient, as it would be to expect them all to become finished musicians if they were taught music.

With such conditions is it any wonder that orthodontia is regarded as more or less of a nuisance to the average practitioner! He avoid this work when he can or reluctantly undertake it, or lets the teeth alone to be straightened by time and nature. Or puts off attempting interference until the patient is past the age for easy and complete treatment. Or resort to the inexcusable practice of extracting for regulating. Hundreds of dentists have told me they dreaded the work and avoided it when possible, and one dentist told me he had as soon see his satanic majesty coming as a case of crooked teeth.

A busy general practitioner has little time to devote to orthodontia, if he undertakes it he must neglect other practice, for of all dental operations those of orthodotia admit of the least neglect.

If these operations are not carried along positively and quickly discouragement and failure are the inevitable result, bringing discredit not only to the operator but to the calling as well.

Orthodontia is too broad and complex and too intensely exacting in its requirements to be given a position second to all else in dental training and practice.

Orthodontia is a specialty not only in training but essentially in practice. Whoever would succeed in its practice must love and cultivate art, especially that highest type of art, the human face. He must enthusiastically love his work and strive for the highest ideals. Then will he be willing to work to overcome difficulties, and will have the courage of his convictions and succeed where others would only fail disastrously.

